# The Role of Maternal Serum Ferritin in Gestational Diabetes Mellitus in Southeast Asia

**DOI:** 10.7759/cureus.72155

**Published:** 2024-10-22

**Authors:** Izzatul 'Aliaa Badaruddin, Ariff Norbani, Jing Ya Chin, Ezzah Sofea Md Said, Uvarrajeswari Selvarajoo, Nur Tihani Mohammad Khaidir, Nurul Fahmiza Tumiran, Raja zahratul Azma, Nor Azlin Mohamed Ismail

**Affiliations:** 1 Department of Pathology, Faculty of Medicine, National University of Malaysia, Kuala Lumpur, MYS; 2 Faculty of Medicine, National University of Malaysia, Kuala Lumpur, MYS; 3 Department of Obstetrics and Gynecology, Faculty of Medicine, National University of Malaysia, Kuala Lumpur, MYS

**Keywords:** fasting blood glucose, ferritin, gestational diabetes, haemoglobin, oral glucose tolerance

## Abstract

Introduction

Gestational diabetes mellitus (GDM) poses a significant health concern due to the numerous risks it presents to both the mother and fetus. While serum ferritin typically declines in cases of iron deficiency anemia, which is common during pregnancy, it may increase in the presence of inflammation. This study aims to evaluate the significance of serum ferritin in the context of GDM, considering its multifaceted role in these interconnected conditions.

Methodology

This cross-sectional study included pregnant women attending an obstetrics clinic for an oral glucose tolerance test at Hospital Canselor Tuanku Muhriz in Kuala Lumpur, Malaysia. With consent, demographic data, risk factors for GDM, and blood samples were collected. Blood samples were analyzed for serum ferritin, fasting blood glucose, two-hour post-glucose levels, and full blood count. The assessment of risk factors for GDM and the diagnosis of GDM adhered to Malaysian guidelines. The Mann-Whitney U test was employed to compare variables between the GDM and non-gestational diabetes mellitus (non-GDM) groups. Additionally, univariate and multivariate regression analyses were conducted to investigate the role of serum ferritin in GDM. All analyses were performed using IBM SPSS Statistics for Windows, Version 28.0 (Released 2021; IBM Corp., Armonk, NY, USA), with statistical significance set at p < 0.05.

Results

Among 172 pregnant women with an average gestational age of 23.6 weeks, the incidence of GDM was higher in those with risk factors (84.8%) compared to non-GDM participants (75.5%). Women with GDM had a slightly higher mean serum ferritin level (50.24 ± 29.00 ng/mL) than those without GDM (45.00 ± 58.69 ng/mL). However, ferritin levels did not significantly differ between the GDM and non-GDM groups (p = 0.669) or between those with and without risk factors (p = 0.374). While a higher BMI (OR: 0.832, p = 0.017) and ferritin (OR: 1.022, p = 0.031) levels independently predicted GDM, the predictive value of ferritin was borderline significant when combined with glucose tests (area under the curve = 0.689, p = 0.051).

Conclusions

Higher maternal serum ferritin levels in the mid-trimester are associated with increased BMI and GDM. However, maternal serum ferritin is most effective at predicting the incidence of GDM when used alongside standard glucose measurements, especially in women with elevated BMI.

## Introduction

Gestational diabetes mellitus (GDM) is characterized by glucose intolerance that is identified during pregnancy through established testing protocols. The development of GDM is influenced by various factors, including genetic, hormonal, and environmental elements [1]. These diverse pathways contribute to varying levels of risk for individuals developing GDM.

The intricate interplay between serum ferritin, iron supplementation, and iron levels has become a focal point in pregnancy-related research. While administering iron supplements is a common approach to addressing maternal iron deficiency during pregnancy, recent studies have revealed a more nuanced relationship [2,3]. Advances in research are highlighting a potential link between iron status - particularly serum ferritin levels - iron supplementation, and the onset of GDM [4,5]. Elevated serum ferritin levels in women are associated with an increased risk of GDM, which may be attributed to heightened insulin resistance and increased insulin secretion from pancreatic β-cells, potentially leading to β-cell exhaustion. Additionally, heme iron, known to enhance systemic iron stores, contributes to oxidative stress within pancreatic cells, further exacerbating the pathophysiological cascade associated with GDM [6].

Serum ferritin is regarded as a reliable, noninvasive marker for assessing iron stores in the body and has played a crucial role in understanding iron status. Integrating routine assessments of iron status, including serum ferritin levels, into prenatal care protocols may enable healthcare providers to identify at-risk individuals and implement targeted interventions to mitigate the development or progression of GDM. The complex interplay between iron metabolism, oxidative stress, and insulin resistance underscores the need for further investigation into the role of serum ferritin in the development of GDM. This study aims to examine the role of mid-trimester maternal serum ferritin and various laboratory markers, evaluating the predictive value of serum ferritin for GDM.

## Materials and methods

Study design and sample size calculation

This prospective study was conducted at the Obstetrics and Gynecology Clinic, Hospital Canselor Tuanku Muhriz (HCTM), Malaysia, between May 2022 and December 2023. The sample size was determined based on a serum ferritin study in mid-trimester pregnant women in India by Pandey et al., which reported a mean difference in serum ferritin of 28.82 ng/mL between the GDM and non-gestational diabetes mellitus (non-GDM) groups [7]. Using a t-test, we considered an 80% power and a 95% CI to calculate the sample size.

Ethical approval

The HCTM serves as a teaching hospital for the Faculty of Medicine at Universiti Kebangsaan Malaysia (UKM). This study received approval from the UKM Research Ethics Committee under approval number JEP-2022-248.

Participant recruitment and sample collections

The study involved 172 women with singleton pregnancies at a gestational age between 14 and 29 weeks, who attended the Obstetrics and Gynecology Clinic at HCTM for a modified oral glucose tolerance test (mOGTT). The mOGTT served as a selective screening tool for women at risk of developing GDM, utilizing a 75-gram oral glucose tolerance test (OGTT). Additionally, in Malaysia, pregnant women over 25 years of age without any risk factors are also recommended for GDM screening using the mOGTT [8].

The risk factors for GDM included a pre-pregnancy BMI greater than 27 kg/m², a previous history of GDM, a first-degree relative with diabetes mellitus, a history of macrosomia, poor obstetric history (such as unexplained intrauterine death, congenital anomalies, and shoulder dystocia), repeated episodes of moderate glycosuria, current obstetric concerns (such as essential hypertension, pregnancy-induced hypertension, polyhydramnios), and the use of steroids [9].

Patients were excluded if they had an acute infection or inflammation, were known to be anemic or receiving intravenous iron treatment prior to pregnancy, received a blood transfusion during the current pregnancy, were diagnosed with any chronic conditions prior to pregnancy (including viral hepatitis, alcoholic liver disease, and malignancy), or had overt diabetes.

Consent and medical history were obtained, and blood samples were collected for serum ferritin and full blood count (FBC) during the fasting blood glucose (FBG) and two-hour post-glucose (2-HPG) tests. Women attending the mOGTT without any risk factors for GDM other than being 25 years of age or older were classified as the no-risk group. In contrast, women of any age presenting with one or more risk factors were categorized as the risk group. GDM status was confirmed if either the FPG was ≥5.1 mmol/L or the 2-HPG was ≥7.8 mmol/L, or both [8].

Laboratory analysis

All samples were analyzed immediately upon receipt at the laboratory. Blood samples for FBC, serum ferritin, FBG, and 2-HPG tests were collected in potassium ethylene diamine tetraacetic acid, serum separator, and fluoride tubes, respectively.

The FBC was performed using flow cytometry with a semiconductor laser method and the sodium lauryl sulfate hemoglobin method. It included measurements of hemoglobin level, red blood cell count, hematocrit, white blood cell count, red blood cell indices, mean corpuscular hemoglobin, and mean corpuscular volume. Blood samples for serum ferritin were centrifuged at 3,000 revolutions per minute for approximately 10 minutes and subsequently analyzed using the Abbott Architect i2000 analyzer (Abbott, Chicago, IL, USA), based on the chemiluminescent immunoassay technique, with results reported in mmol/L.

FBG and 2-HPG levels were analyzed using the Abbott Architect c8000 and c16000 analyzers (Abbott), which ensured inter-analyzer precision. The laboratory’s plasma glucose measurement principle utilized the hexokinase method, with results also reported in mmol/L.

Data analysis

All data were imported and analyzed using IBM SPSS Statistics for Windows, Version 28.0 (Released 2021; IBM Corp., Armonk, NY, USA). Categorical data were presented as numbers and percentages for participant characteristics, while continuous data were expressed as means and SDs (mean ± SD). The Shapiro-Wilk test was employed to assess data normality before conducting further analyses for comparison and association.

The Mann-Whitney U test was utilized to compare serum ferritin levels between groups for continuous, non-normally distributed data. Additionally, binary logistic regression and receiver operating characteristic (ROC) curve analysis were employed to investigate the relationship and role of maternal serum ferritin levels in GDM. Statistical significance was set at p < 0.05.

## Results

Participant overview

Out of 227 participants recruited, the final analysis included 172 pregnant women, with a mean gestational age of approximately 23 ± 6 weeks and a mean age of 33.0 ± 4.8 years. The overall BMI of the recruited population was 26.7 ± 5.7 kg/m². A higher percentage of individuals with risk factors was observed in the GDM group (84.8%) compared to the non-GDM group (75.5%).

In terms of laboratory analysis, the GDM group exhibited slightly higher serum ferritin levels (50.24 ± 29.00 µg/mL) compared to the non-GDM group (45.00 ± 58.69 µg/mL). Additionally, the GDM group demonstrated higher FBG levels and significantly elevated 2-HPG levels (8.43 ± 1.62 mmol/L) compared to the non-GDM group (5.56 ± 1.03 mmol/L), highlighting notable differences in glucose metabolism between the two groups (Table 1).

**Table 1:** Populations overview ^#^ Mean and SD 2-HPG, two-hour post-glucose; FBG, fasting blood glucose; GDM, gestational diabetes mellitus; non-GDM, non-gestational diabetes mellitus

Characteristic	Overall (n = 172)	GDM (n = 33)	Non-GDM (n = 139)
Demographic data			
Age (years)^#^	33.0 + 4.8	33.6 + 4.9	32.8 + 4.8
BMI (kg/m²)^#^	26.7+ 5.7	26.6 + 5.6	26.8 + 5.8
Gestational age (weeks)^#^	23.7 + 6.0	23.2 + 6.4	23.8 + 5.9
Individuals with risk factors (n (%))	133 (77.3)	28 (84.8)	105 (75.5)
Laboratory analysis^#^			
Serum ferritin levels (ng/mL)	46.00 + 57.90	50.24 + 29.00	45.00 + 58.69
Hemoglobin (g/dL)	11.53 + 1.03	11.48 + 1.02	11.33 + 1.03
FBG levels (mmol/L)	4.40 + 0.43	4.77 + 0.62	4.31 + 0.33
2-HPG levels (mmol/L)	6.10 + 1.62	8.43 + 1.62	5.56 + 1.03

Maternal serum ferritin in the presence of risk factors and GDM

The Shapiro-Wilk test indicated that the data were not normally distributed. Consequently, the Mann-Whitney U test was used to compare the two groups, revealing no significant difference in the median and IQR (median (IQR)) of maternal serum ferritin levels between the GDM group (28.96 (36.73)) and the non-GDM group (31.93 (38.29); p = 0.669). Additionally, there was no significant difference between those with risk factors for GDM (33.21 (35.25)) and those without risk factors (28.61 (49.82); p = 0.374) (Table 2).

**Table 2:** Comparison between groups * Median (IQR) ^*^ p-value <0.05 is considered significant ^#^ Mann-Whitney U test GDM, gestational diabetes mellitus; non-GDM, non-gestational diabetes mellitus

Variables	Serum ferritin levels (ng/mL)	Z-statistic	p-value^#*^
GDM	28.96 (36.73)	0.428	0.669
Non-GDM	31.93 (38.29)		
Risk group	33.21 (35.25)	1.176	0.374
No risk group	28.61 (49.82)		

The relationship of maternal serum ferritin with other variables in predicting GDM

The univariate analysis revealed that BMI and maternal serum ferritin independently and significantly predicted the occurrence of GDM, with ORs of 0.832 (95% CI: 0.716-0.968; p = 0.017) and 1.022 (95% CI: 1.002-1.041; p = 0.031), respectively. An OR of 0.832 suggests that a higher BMI is associated with lower odds of developing GDM, while an OR of 1.022 indicates that higher maternal serum ferritin is linked to an increased likelihood of GDM. FBG and 2-HPG levels emerged as the strongest predictors of GDM, with ORs of 313.084 (95% CI: 11.888-8254.298; p < 0.001) and 45.957 (95% CI: 8.348-252.997; p < 0.001), respectively. In the multivariate analysis, the inclusion of BMI improved the predictive value, yielding an OR of 0.189 (95% CI: 0.717-0.956; p = 0.010). However, maternal serum ferritin was not statistically significant, with a p-value of 0.074. These findings suggest that incorporating maternal serum ferritin into the assessment adds no value beyond the established FBG and 2-HPG measurements (Table 3, Table 4).

**Table 3:** Univariate binary logistic regression analysis ^*^ p-value <0.05 is significant ^** ^p-value <0.001 is strongly significant FBG, fasting blood glucose; 2-HPG, two-hour post glucose

Variable	B	OR	95% CI	p-value
Age	0.085	1.089	0.914-1.298	0.339
BMI (kg/m^2^)	-0.184	0.832	0.716-0.968	0.017*
Ferritin (µg/L)	0.021	1.022	1.002-1.041	0.031*
FBG (mmol/L)	4.869	313.084	11.888-8254.298	<0.001**
2-HPG (mmol/L)	3.361	45.957	8.348-252.997	<0.001**

**Table 4:** Multivariate binary logistic regression analysis ^*^ p-value <0.05 is significant ^**^ p-value <0.001 is strongly significant 2-HPG, two-hour post glucose; FBG, fasting blood glucose

Variable	B	OR	95% CI	p-value
Step 1				
2-HPG (mmol/L)	2.232	9.321	4.167-20.848	<0.001**
Step 2				
FBG (mmol/L)	3.63	37.698	4.258-333.760	<0.001**
2-HPG (mmol/L)	3.075	21.66	5.579-84.091	
Step 3				
BMI (kg/m^2^)	-0.189	0.828	0.717-0.956	0.010*
FBG (mmol/L)	4.869	130.146	8.361-2,025.803	<0.001**
2-HPG (mmol/L)	3.361	28.806	6.527-127.131	<0.001**

The value of maternal serum ferritin in GDM

**Figure 1:**
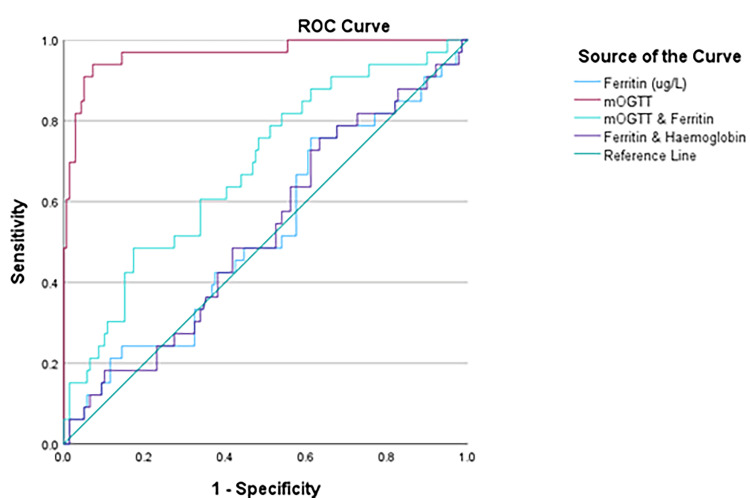
ROC curve mOGTT, maternal oral glucose tolerance test; ROC, receiver operating characteristic

The ROC curve analysis indicates that the mOGTT is an excellent predictor for GDM, with an area under the curve (AUC) of 0.967 (p < 0.001), confirming its status as the gold standard for diagnosis (Figure 1, Table 5). In contrast, serum ferritin alone and in combination with hemoglobin demonstrate poor discriminatory power, with AUCs around 0.524, suggesting ineffectiveness in predicting GDM. Although combining mOGTT and ferritin results in a modest improvement in predictive ability with an AUC of 0.689 (p = 0.051), this combination still does not surpass the predictive accuracy of mOGTT alone. Therefore, while mOGTT remains the most effective diagnostic tool, the addition of ferritin offers limited value in this context.

**Table 5:** Predictive value of maternal serum ferritin in GDM ^*^ p < 0.05 is significant ^** ^p < 0.001 is strongly significant GDM, gestational diabetes mellitus; mOGTT, maternal oral glucose tolerance test; non-GDM, non-gestational diabetes mellitus

Variable	Area	95% CI	p-value
mOGTT	0.967	0.932-1.000	0.000**
mOGTT and ferritin	0.689	0.590-0.788	0.051
Ferritin	0.524	0.413-0.635	0.669
Ferritin and hemoglobin	0.524	0.413-0.633	0.663

## Discussion

The precise mechanisms connecting elevated serum ferritin to GDM remain unclear. However, it is hypothesized that iron overload may contribute to insulin resistance and impaired glucose tolerance through mechanisms such as oxidative damage to pancreatic beta cells and interference with insulin's ability to suppress hepatic glucose production [10].

Several studies have suggested that elevated serum ferritin levels can identify individuals at higher risk for developing GDM. For instance, Bowers et al. found a significant association between higher plasma ferritin levels in early pregnancy and increased risk of GDM, even after adjusting for key risk factors like pre-pregnancy BMI and advanced maternal age [11]. Our study partially aligns with these findings, as we also observed higher mean maternal serum ferritin levels in the GDM group compared to the non-GDM group; however, this was not the case when serum ferritin was expressed as a median. While Bowers et al. reported higher mean ferritin levels in the GDM group, they did not provide median values. Given the large sample size in their study, the potential influence of outliers on their mean ferritin values cannot be discounted. Nonetheless, we found maternal serum ferritin to be a poor predictor of GDM incidence compared to their findings. Several factors may account for these discrepancies, including the effect of outliers on mean ferritin values, variations in population characteristics, differences in GDM diagnostic criteria, and the timing of ferritin measurements during pregnancy.

Interestingly, our study revealed a notable discrepancy between the mean and median maternal serum ferritin levels. Although the mean serum ferritin was higher in the GDM group, the median level was higher in the non-GDM group. This prompted a closer examination of individual patient data for potential outliers. Specifically, participants with elevated serum ferritin levels in the non-GDM group exhibited multiple risk factors for GDM, such as a strong family history and a prior diagnosis of GDM. Malaysian clinical guidelines recommend repeating the OGTT in the third trimester for high-risk individuals, even if the initial OGTT is normal [8]. Unfortunately, this participant was unable to follow up after her first OGTT.

Similar to our findings, Soheilykhah et al. reported elevated serum ferritin levels in pregnant women with GDM [12]. However, unlike their study, we did not find a statistically significant difference in ferritin levels between the GDM and non-GDM groups. This discrepancy may stem from differences in the study populations. Soheilykhah et al. focused on women with hemoglobin levels above 12.0 g/dL, while our study included participants with hemoglobin levels closer to anemia [12]. Additionally, they recruited women in their first trimester (12-16 weeks), whereas our study primarily involved women in their second trimester, during which physiological changes like increased blood volume can accentuate pregnancy-associated dilutional anemia. These variations in iron status and gestational age may help explain the differences in findings.

In contrast, Zhang et al. found that elevated ferritin levels in early pregnancy were linked to higher HbA1c, FBG levels, and an increased risk of insulin resistance later in pregnancy [13]. The ethnic diversity and higher prevalence of metabolic dysregulation in our study population could account for this discrepancy. Moreover, the timing of our ferritin measurements, likely occurring before the onset of physiological anemia, might have influenced our results.

While our study also assessed serum ferritin levels during the second trimester, it differs from the prospective observational study by Pandey et al. in a key aspect: our analysis focused on a population with a different baseline hemoglobin profile than Pandey’s study, which specifically enrolled non-anemic pregnant women [7]. This difference in participant characteristics may contribute to the contrasting findings. Additionally, while Pandey et al. provide valuable insights through their analysis using mean serum ferritin levels [7], our study utilized statistical methods that account for the distribution of individual data points, allowing for a comprehensive assessment of the potential influence of outliers on the observed relationship between serum ferritin and GDM risk. Finally, our study indicated that the relationship between maternal serum ferritin and GDM diagnosis is indirectly linear (p = 0.074), while Sun et al., in their meta-analysis, revealed a linear relationship (p = 0.061) between maternal serum ferritin and GDM [14].

Despite our contrasting findings, we contribute to the ongoing discussion regarding the relationship between serum ferritin and the risk of GDM. While our results did not reveal a significant association, which contrasts with some previous reports, our study benefits from mid-trimester ferritin measurement and a diverse study population. However, the limitations of relying on a single ferritin assessment and the lack of comprehensive iron status data should be acknowledged. Future longitudinal studies incorporating detailed assessments of iron metabolism, genetic analyses, and subgroup analyses are warranted to elucidate the complex interplay between iron status and GDM development.

## Conclusions

While the OGTT remains the gold standard for diagnosing GDM in our population, our study did not find a statistically significant association between serum ferritin levels and the development of GDM. Nonetheless, this research offers valuable insights into the role of pre-pregnancy BMI in the ongoing exploration of GDM risk factors.

Future longitudinal studies involving pregnant women with and without anemia could further clarify the relationship between maternal serum ferritin, iron status, and GDM. Additionally, providing more information on mid-trimester maternal serum ferritin levels could enhance personalized iron therapy prescriptions, potentially minimizing the risk of GDM. Such insights could improve risk assessment and guide more tailored prevention and management strategies.
